# An Antibiotic-Loaded Silicone–Hydrogel Interpenetrating Polymer Network for the Prevention of Surgical Site Infections

**DOI:** 10.3390/gels9100826

**Published:** 2023-10-19

**Authors:** Rasmus Birkholm Grønnemose, Ditte Rask Tornby, Sara Schødt Riber, Janni Søvsø Hjelmager, Lars Peter Schødt Riber, Jes Sanddal Lindholt, Thomas Emil Andersen

**Affiliations:** 1 Department of Clinical Microbiology, Odense University Hospital, 5000 Odense, Denmark; rasmus.birkholm.gronnemose@rsyd.dk (R.B.G.); dstornby@health.sdu.dk (D.R.T.); 2 Research Unit of Clinical Microbiology, University of Southern Denmark, 5000 Odense, Denmark; jhjelmager@health.sdu.dk; 3 Department of Cardiothoracic and Vascular Surgery, Odense University Hospital, 5000 Odense, Denmark; sara.schodt.riber@rsyd.dk (S.S.R.); lars.riber@rsyd.dk (L.P.S.R.); jes.sanddal.lindholt@rsyd.dk (J.S.L.); 4 Research Unit of Cardiothoracic and Vascular Surgery, University of Southern Denmark, 5000 Odense, Denmark

**Keywords:** *Staphylococcus aureus*, infection, antibacterial hydrogel, interpenetrating polymer network, surgical site infection

## Abstract

Surgical site infections (SSIs) are among the most frequent healthcare-associated infections, resulting in high morbidity, mortality, and cost. While correct hygiene measures and prophylactic antibiotics are effective in preventing SSIs, even in modern healthcare settings where recommended guidelines are strictly followed, SSIs persist as a considerable problem that has proven hard to solve. Surgical procedures involving the implantation of foreign bodies are particularly problematic due to the ability of microorganisms to adhere to and colonize the implanted material and form resilient biofilms. In these cases, SSIs may develop even months after implantation and can be difficult to treat once established. Locally applied antibiotics or specifically engineered implant materials with built-in antibiotic-release properties may prevent these complications and, ultimately, require fewer antibiotics compared to those that are systemically administered. In this study, we demonstrated an antimicrobial material concept with intended use in artificial vascular grafts. The material is a silicone–hydrogel interpenetrating polymer network developed earlier for drug-release catheters. In this study, we designed the material for permanent implantation and tested the drug-loading and drug-release properties of the material to prevent the growth of a typical causative pathogen of SSIs, *Staphylococcus aureus*. The novelty of this study is demonstrated through the antimicrobial properties of the material in vitro after loading it with an advantageous combination, minocycline and rifampicin, which subsequently showed superiority over the state-of-the-art (Propaten) artificial graft material in a large-animal study, using a novel porcine tissue-implantation model.

## 1. Introduction

Surgical site infection (SSI) is a common nosocomial infection associated with considerable morbidity and mortality. According to the World Health Organization, SSIs are the most frequent type of healthcare-associated infections (HAIs) in low- and middle-income countries, and the second most common type of HAIs in Europe and the USA after urinary tract infections [1]. Depending on the type of surgery, SSIs are often caused by skin bacteria, such as *Staphylococcus aureus* (*S. aureus*), and coagulase-negative staphylococci that enter the surgical wound during or after surgery [2,3]. The risk of post-surgery infectious complications is influenced by several factors related to the patient and the specific procedure used, and extensive guidelines have been formulated with recommended preventative measures [1,4]. In addition to thorough hygiene protocols, an important recommended measure for the prevention of SSIs is the use of antibiotic prophylaxis [5]. The rise in antibiotic resistance among pathogens typically responsible for SSIs, however, has increasingly complicated prophylactic antimicrobial therapy [6]. Moreover, the overuse or misuse of antibiotic prophylaxis and general inappropriate antimicrobial stewardship may induce antibiotic resistance in bacteria colonizing a patient, further exacerbating the problem [3,7]. The result is the extended duration of systemic antibiotic therapy, more frequent surgical revision, and increased morbidity and mortality [8].

Antibiotic prophylaxis is particularly indicated for procedures involving foreign-body implantations, such as prosthetic cardiac valves, prosthetic vascular grafts, and orthopedic devices [3]. Surgical procedures involving the implantation of foreign materials increase the risk of infection as they provide an inert and non-responsive surface for bacteria to adhere to, which, undetected by the host, initiate the colonization of the device or implant and form biofilms [9]. Such progression may occur even in situations where systemic antibiotic prophylaxis is used, e.g., during periods of time when the antibiotic being used reaches subinhibitory concentrations, such as in between oral and intravenous administrations, or when the site of infection is poorly vascularized or, in other ways, presents a limited availability to the antibiotic. The resulting biofilm provides a physical barrier that protects the pathogen against the host immune response and antibiotics, and the close proximity of bacteria in the biofilm may further promote resistance development [9,10]. To prevent SSIs associated with implants, the local administration of antibiotics, e.g., through slow release of antibiotics from the material or device itself, can be an ideal way to prevent bacteria from colonizing the material. This may reduce the risk of the formation of resilient biofilms at or near the device, and local administration requires much less total antibiotics to reach local inhibitory concentrations compared to systemic administration, thus preventing side effects and the induction of antibiotic resistance in bacteria elsewhere in the body, such as in the intestinal microbiota [11].

In earlier work, we explored the potential of modifying silicone rubber with a hydrogel to obtain antimicrobial properties by integrating these two materials into so-called interpenetrating polymer networks (IPNs). To produce these IPNs, the silicone part is swelled in supercritical CO_2_, and a hydrogel is polymerized de novo inside the silicone scaffold, essentially forming a composite material that holds advantageous material properties from both parts [12,13]. These IPN materials have shown promising properties for storing and releasing antibiotics and other drugs [14,15], and we successfully tested them as functional intravenous catheters for the prevention of the bacterial colonization of catheters inserted into the bloodstream in a large-animal model [15].

In the current study, we further investigated the latest iteration of the silicone–hydrogel IPN, containing 2-hydroxyethyl methacrylate co-polymerized with ethylene glycol methyl ether acrylate, which improves the flexible properties and mechanical strength of the material [14]. We speculated that it could be used in permanently tissue-implanted materials such as vascular grafts, which are used in patients to provide an access site to the bloodstream, e.g., for hemodialysis [16]. This function places higher demand on the material as it needs to have mechanical properties that mimic those of tissue and blood vessels, and it must be blood- and tissue-compatible to reduce adverse host responses and rejection. Currently used vascular grafts suffer from a variety of complications with regard to, e.g., infection. These state-of-the-art grafts do not use antimicrobial loadings/coats, which therefore require prophylactics and/or post-operative antibiotics. However, this is not always enough and, in addition, infections can occur after systemic treatment as this treatment cannot continue indefinitely. Additionally, the unnecessary use of broad-spectrum agents may exacerbate local resistance profiles [8,17]. Lastly, patients in need of a vascular graft are often immunologically compromised and, thus, susceptible to infection, emphasizing the need for additional measures to prevent SSIs in this patient group.

In this study, we assessed the antibacterial properties of the material when loaded with minocycline and rifampicin, which is a combination that has advantageous properties, especially against typical SSI pathogens, such as *S. aureus*. The loading and release properties of the material were analyzed using in vitro assays. Moreover, the antimicrobial properties of the material were tested in vivo in a large-animal model of implant-associated SSIs in pigs, developed specifically for this study.

## 2. Results

### 2.1. Drug-Release Quantification

UV-vis spectra covering 190 nm to 600 nm were recorded for minocycline, rifampicin, and the two drugs in a 1:1 (*w*/*w*) mixture to identify local absorption extrema (Figure S1a). At wavelength λ = 245 nm, rifampicin and minocycline had a local minimum and a local maximum, respectively. At wavelength λ = 475 nm, rifampicin showed an absorption maximum. At λ = 245 nm and λ = 475 nm, different antibiotic concentrations were measured to create calibration graphs (Figure S1b). For the calibration curves, the known standard concentrations were aligned with the measured absorbance intensities and a linear regression curve was formed.

To determine the concentration of released antibiotics from the IPN material, the equations from the calibration graphs’ linear regression were used. The concentration of rifampicin in the release medium was calculated using the equation for pure rifampicin at λ = 475 and the absorbance measured at that wavelength. The concentration of minocycline in the release medium was calculated using Equations (1)–(3). The theoretical absorbance of rifampicin (Rif_abs_(λ245)) was calculated using the standard curve equation for pure rifampicin at 245 nm and the calculated concentration of rifampicin at 475 nm (Rif_calc_(λ475)) (Equation (1)). To calculate the absorbance of minocycline (Mino_abs_(λ245)) in the release medium, the calculated Rif_abs_(λ245) was subtracted from the absorbance measured at 245 nm for the release medium (RM_abs_(λ245)) (Equation (2)). Mino_abs_(λ245) was subsequently used in the standard curve equation for minocycline at λ = 245 to calculate the concentration of minocycline (C_mino_) (Equation (3)).

Rif_abs_(λ245) = 0.01589 × Rif_calc_(λ475) + 0.03488 (1)

Mino_abs_(λ245) = RM_abs_(λ245) − Rif_abs_(λ245) (2)

C_mino_ = (Mino_abs_(λ245) − (−0.009051))/0.01589 (3)

**Figure 1:**
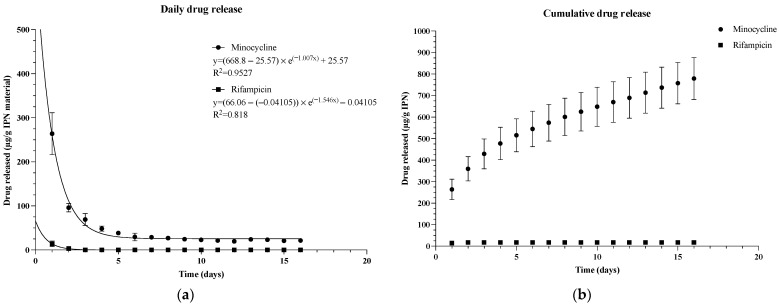
(**a**) Daily release and (**b**) cumulative release of minocycline and rifampicin from the IPN material. The data points indicate µg of drug released per gram of IPN material measured daily. Data are shown as mean values of four samples ± standard deviation over a time span of 16 days.

An initial burst release of 263.8 ± 47.5 µg minocycline per gram of IPN was observed on day 1 (Figure 1a). In the following days, the daily release changed from 96.0 ± 9.6 µg/g IPN to a steadier 29.5 to 19.6 µg/g IPN 14 days. The release of rifampicin was observed to be much lower than that of minocycline, with 14.0 ± 6.4 µg/g IPN being released on day 1. Thereafter, the daily release of rifampicin decreased to 3.2 ± 3.8 µg/g IPN before the release became 0 on day 3 (Figure 1a). Over a time span of 16 days, the total release of minocycline and rifampicin from the IPN material reached 779.3 ± 97.6 and 17.3 ± 9.7 µg/g of IPN, respectively (Figure 1b). The daily drug release is best explained through an exponential decay model, as the rate at which the drugs were released was proportional to the amount that was left in the IPN material. The release of minocycline and rifampicin both followed a one-phase decay model (R^2^ = 0.953 and R^2^ = 0.818, respectively) (Figure 1a). The percentual daily drug release is shown in Figure S2.

### 2.2. Functional Release Assay

The biological efficacy of the antibiotic released from the IPNs was tested against *S. aureus* in functional release assays. First, the minimum inhibitory concentration (MIC) and minimum bactericidal concentration (MBC) for the antibiotics were determined. Both minocycline and rifampicin had MICs of ≤0.44 µg/mL, while the minocycline and rifampicin in a 1:1 (*w*/*w*) mixture (hereafter M/R) had a MIC of ≤0.22 µg/mL. The MBCs of minocycline, rifampicin, and the M/R mixture were 3.52 µg/mL, ≤0.44 µg/mL, and ≤0.22 µg/mL, respectively.

To assess the growth-inhibitory effect of the released antibiotics, bacteria were added to the release media diluted in 1:1 growth media, and growth was visually assessed after overnight incubation. No visual growth was observed in any of the release medium samples collected over the 16-day period, indicating that released antibiotics reached values greater than the MIC values (Figure S3).

**Figure 2:**
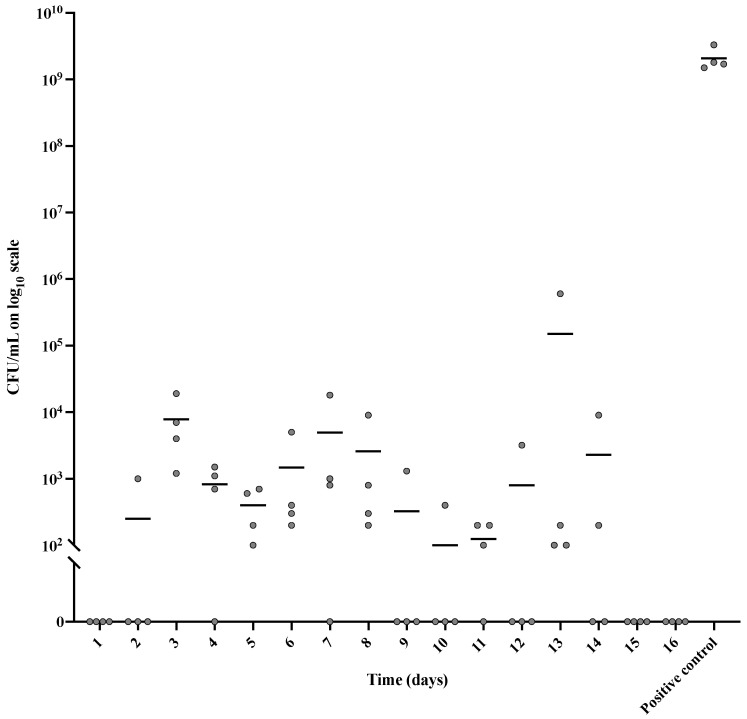
Bactericidal effect of antibiotics released from M/R-loaded IPN patches and positive controls, estimated based on CFU counts. The lower detection limit is 100 CFU/mL. Data are shown as mean values (black bars) and as data points for individual patches per day over a period of 16 days. The positive control was only determined on day 1. All groups showed significantly lower CFU counts compared to the positive control (*p* < 0.0001). The groups were compared using one-way ANOVA with Tukey’s Honest Significant Difference test.

The release medium was further tested for bactericidal effect against *S. aureus*. This was measured as the reduction in colony-forming units (CFU) of *S. aureus* exposed to the release medium (Figure 2). The release medium from the IPN material with no drugs loaded was tested as a negative control to ensure that the effect seen was due to antibiotic release from the IPN material. No bacterial reduction was observed when testing the antibiotic-free release medium.

The release media reduced viable bacteria by between 97.5% and 100% compared to the inoculum (Figure 2). For all replicates over the period of 16 days, the growth of *S. aureus* was inhibited significantly by a minimum of 97.5%, with only days 3 and 13 showing inhibition less than 99.9%.

### 2.3. Agar Disc Diffusion Assay

**Figure 3:**
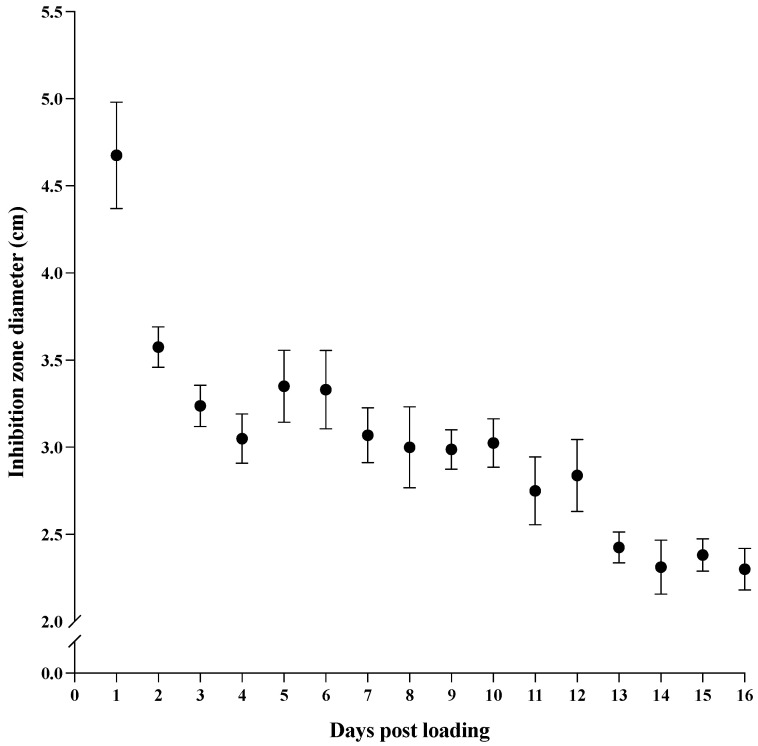
Agar disc diffusion assay with rifampicin- and minocycline-loaded IPN discs on *S. aureus*-inoculated agar plates. Data are shown as the means of four samples ± standard deviation over a time span of 16 days.

The inhibiting effect near the surface of the antibiotic-loaded IPN patches against *S. aureus* was estimated by inhibition zone measurements on inoculated agar plates over a time span of 16 days. All replicates showed an inhibition zone throughout the period of 16 days (Figure 3; examples of inhibition zones are shown in Figure S4). Starting with a mean inhibition zone of 4.7 cm, the zone decreased to a steady zone diameter ranging between 3.6 and 2.3 cm from day 2 to day 16. No inhibition zones were observed for the control patches without loaded antibiotics (Figure S5). Overall, this result indicates that antibiotics retain their antibiotic effect while being bound to and released from the IPN material, suggesting prolonged release and local efficacy.

### 2.4. Porcine Surgical Site Infection Model

To assess the performance of the M/R-loaded IPN patches in preventing surgical site infections, we developed a porcine surgical site infection model using a design where the M/R-loaded IPN patches could be tested against ePTFE patches; the latter is currently used for implantable vascular grafts in humans.

**Figure 4:**
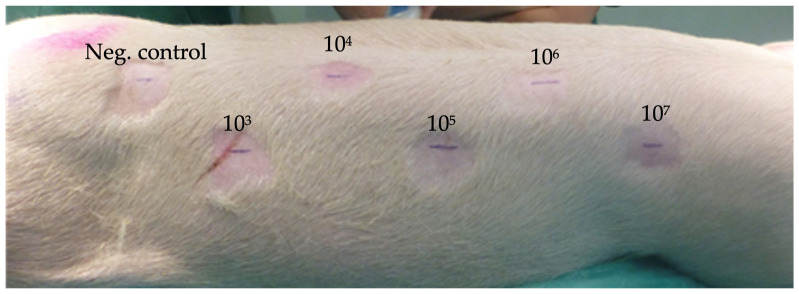
Illustration of the incision marks and distribution of the patches on one side of the pig. Inocula in colony-forming units added to each incision site is indicated in red. The same distribution was applied on the other site of the pig. Each pig was implanted with six ePFTE patches on one site and six IPN patches on the other site.

A preliminary experiment was performed with an infective dose escalation of *S. aureus* to establish the minimum required number of bacteria to cause a persistent surgical site infection in all six pigs after 2 weeks and to assess any indication of SSI prevention in the M/R-loaded IPN patches vs. the ePFTE patches. The experiment was performed in six pigs, each of which was implanted with six ePFTE patches on one site and six IPN patches on the other site. Prior to wound closure, the patches were inoculated with increasing concentrations of *S. aureus* from 10^3^ to 10^7^ CFU (Figure 4).

**Figure 5:**
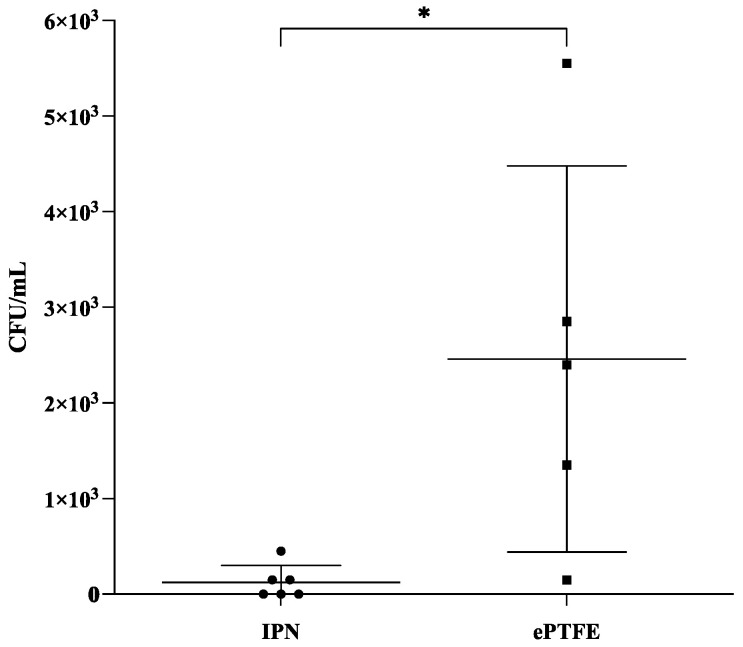
Number of implant-associated bacteria after 2 weeks of infection in a porcine tissue implantation model using bacterial dose escalation. Data are shown as means ± standard deviation with individual data points. Only results from the 10^6^ CFU dose are shown. *n* = 5 for the ePTFE patches and *n* = 6 for the M/R-loaded IPN patches. One replicate of the ePTFE patches was excluded due to streptococci contamination. The detection limit is 50 CFU/mL. The groups were compared using Mann–Whitney unpaired *t*-test. * *p* < 0.05.

Infection in 6/6 pigs occurred at the ePTFE patches inoculated with a dose of 10^6^ CFU (Figure 5), while only 2/6 pigs had quantifiable bacteria at the highest dose (10^7^ CFU) for the M/R-loaded IPN patches. Furthermore, significantly fewer CFU were isolated from the 10^6^ inoculated M/R-loaded IPN patches than from the corresponding ePTFE patches inoculated with this inoculum (Figure 5, *p* < 0.05).

**Figure 6:**
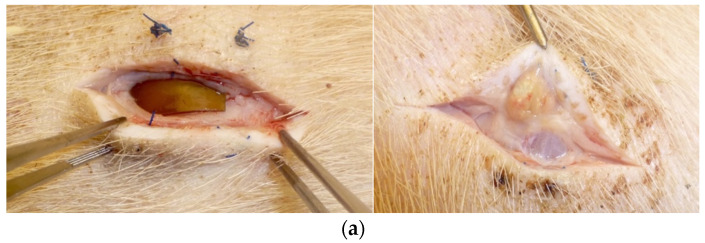

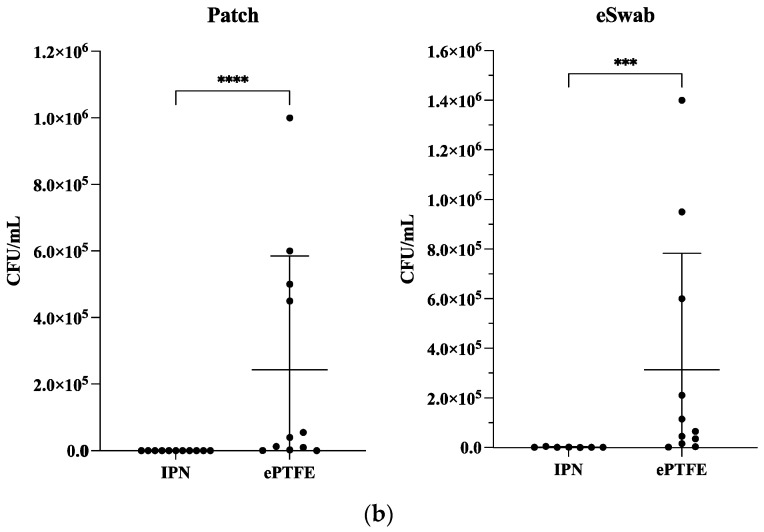
(**a**) Example of a surgical site pocket with an IPN patch (**left**) and an ePTFE patch (**right**) 14 days after inoculation with an infectious dose of 10^6^ CFU. (**b**) Implant-associated bacteria (**left**) and bacteria associated with the surgical site pocket (**right**) after 14 days of infection in the porcine tissue implantation model using a fixed infectious dose of 10^6^ CFU. The detection limit is 50 CFU/mL. Data are shown as means ± standard deviation with individual data points. *n* = 11 for the ePTFE patches and *n* = 12 for the M/R-loaded IPN patches. The groups were compared using Mann–Whitney unpaired t-test. *** *p* < 0.001 and **** *p* < 0.0001.

Following the preliminary experiment, we performed a new porcine tissue implantation experiment in two pigs to test if the M/R-loaded IPN materials, besides preventing growth of implant-associated bacteria, also prevented growth in the surgical pocket. In this experiment, only the 10^6^ CFU dose was used, which had been shown to cause persistent infection on all ePTFE patches in the preliminary experiment. The data obtained for this experiment showed a significant reduction in both the number of implant-associated bacteria, as also seen in the preliminary experiment (*p* < 0.0001), and in the number of bacteria residing in the surgical pocket, as assessed through swab sampling (*p* = 0.0003) (Figure 6).

## 3. Discussion

Contrary to chemical blends or co-polymers, an interpenetrating polymer network (IPNs) is defined as an elastomer composed of two chemically distinct networks that coexist by being physically cross-linked through interlocked polymer chains [18,19]. Each of the polymer components in the network retains the unique properties of the pure polymer; therefore, IPNs offer an advantageous combination of properties, such as high chemical stability combined with good biocompatibility, nontoxicity, and bioavailability [20]. The unique properties of IPNs have enabled their use in various drug-delivery applications, such as drug-carrier systems, microscale capsule-based formulations, and biodegradable implants [20,21,22]. Moreover, we have reported that the drug-release and drug-diffusion properties in silicone–hydrogel IPNs could be used in vascular catheter materials for the prevention of infections [15] and even in the balloon of Foley-type catheters, where an IPN was used as balloon material to enable and control the release of a drug over the balloon wall [23].

In this regard, IPNs offer a conceptual alternative to coatings, which are often difficult to apply to highly chemically stable materials that are typically used in permanent implants, such as silicone. Moreover, an antibiotic drug coating constitutes the material–tissue interface, which may affect how well the device is tolerated in the tissue. In this case, the IPN itself makes up the interphase in contact with the surrounding tissue, and the drug, such as an antibiotic, is released from a reservoir in the bulk of the material. This potentially increases the tissue compatibility of the device.

In earlier reports, IPNs showed promising properties for co-loading and releasing antimicrobial agents over a given period [14,15,23]. In this study, we assessed the antibacterial properties of minocycline and rifampicin impregnated into a silicone–hydrogel IPN. The combination of minocycline and rifampicin has advantageous properties, especially against the typical SSI pathogen *Staphylococcus aureus* (*S. aureus*). Resistance against rifampicin in staphylococcus-associated open wound infections, such as vascular graft infections, is a growing concern and is especially considered a risk that complicates antibiotic prophylaxis, which is common after procedures that involve foreign-body implantations [3,6,7,24]. To overcome this issue, several studies have investigated treatments involving rifampicin accompanied by a second drug, as well as treatments administered locally instead of systemically. Systemic treatment often requires higher doses and it is more prone to lead to antimicrobial resistance [8,11,24]. The advantage of combining rifampicin with minocycline is that it potentially provides long-acting, broad-spectrum bacteriostatic activity. Additionally, minocycline has previously been proven to be a good candidate for incorporation into microcapsules used for the local prevention and treatment of periodontitis [25,26,27].

In our study, the general loading capacity of the antimicrobials in the IPN material was found to be relatively low. The accumulated, combined release of minocycline and rifampicin over a period of 16 days was approximately 796.6 µg of antibiotic per gram of IPN—a relatively small fraction of the 10 mg antibiotics per milliliter used in the drug loading solutions. For rifampicin, the general release was considerably lower than that of minocycline, reaching levels undetectable by UV-vis after two days of release in vitro. However, the proportion of drug released from the material demonstrated antibacterial properties during the entire period. The low release of rifampicin compared to that of minocycline is hypothesized to be due to the equilibrium release during the loading protocol of the IPN material. Despite that, the overall total release value was greater than the MIC value and also greater than the MBC value during the entire period. Although the release medium contained bactericidal antibiotic quantities, the equilibrium release was accounted for when loading the IPN material for subsequent in vivo tests. The initial total burst release of M/R on day 1 was 64.7% of the overall antimicrobials released, which can be considered favorable in situations where an implant is contaminated during the surgical procedure and immediate bacterial clearing is desirable. The subsequent lower, but steady and prolonged, release period, which could potentially last for several weeks, according to our previous study [15], may retain an effect against bacteria later entering the site through hematogenous spreading or via penetration from the skin surface. This long-term antibiotic release after the burst release could result in proper resilience against bacterial contaminants and device-related infection post-surgery. A prolonged release with a release duration past the initial graft implantation should improve outcomes, with less infections arising from the continuous contamination of the surgical site and from the systemic spread of bacteria to the implanted graft.

The antibiotic release medium was further tested for its effect against *S. aureus*. This was measured as the bactericidal effect of the release medium compared to the release medium of the IPN material with no drugs loaded. For all replicates over the period of 16 days, viable *S. aureus* was reduced significantly by a minimum of 97.5%, with only day 3 and 13 showing a reduction of less than 99.9% of *S. aureus*. This result supports the UV-vis data, which showed a total release of antibiotics around or beyond the MIC and MBC values for all release media. For the inhibition-zone measurements of M/R-loaded IPN patches during the 16-day trial period, the zone diameters ranged from 4.7 cm to 2.3 cm. No inhibition zones were observed for the control patches without loaded antibiotics. Overall, this indicates that the antibiotics retain their antibacterial effect while being bound to and released from the IPN matrix, and suggests prolonged local efficacy.

In a previous study, Gao et al. [28] evaluated whether implanted grafts in pigs inoculated with *S. aureus* could be managed through local and systemic antibiotic treatments. In their study, a swab sample from the surface tissue at the surgical site was collected for bacteriological examination. Despite treating the infection with a single dose of local rifampicin, followed by 14 days of systemic rifampicin treatment of *S. aureus*, the graft was still infected upon termination of the experiment. These findings suggest that a longer treatment time is needed, preferably in combination with another antibiotic drug. The combination-loaded IPN tested in the current study may account for this by co-loading with minocycline and using local release that might circumvent problems with too-early clearing of the drug or reduced drug penetration. In our study, the release of antibiotics showed promising results with a significant reduction in *S. aureus* growth in both the number of implant-associated bacteria and the number of bacteria residing in the surgical pocket. Even though the sample size is small and larger-scale preclinical evaluation is needed, the in vitro and in vivo results presented here indicate that the tested silicone–hydrogel IPN material holds potential as an implant material with drug-release properties that enable the prevention of SSIs.

## 4. Conclusions

In conclusion, we investigated the antibacterial performance of a silicone–hydrogel interpenetrating polymer network impregnated with a combination of minocycline and rifampicin. The high combined burst release on day 1 and the subsequent continuous release of antimicrobial agents were found to inhibit the growth of *S. aureus* by a minimum of 97.5% or more for over 16 days, indicating a potential to prevent SSIs that may develop days and perhaps months after implantation. Despite the relatively low loading capacity, the antibacterial properties remained above the M/R MIC level throughout the 16 days and above or around the M/R MBC level throughout, with the exception of 2 days. Additionally, a novel skin-pocket SSI model in pigs was developed and used to test the drug-loaded IPNs. The results from this model supported the in vitro results, showing that both implant-associated bacteria and bacteria associated with the surgical site pocket were significantly lower using the M/R-loaded IPN patches compared to a state-of-the-art heparin-coated ePTFE graft material. While showing promising fundamental drug-release properties in vitro and in vivo, the IPN material tested here is still in an early experimental stage and needs to be tested additionally, e.g., for suitability for replacing blood vessels and to be tested for tissue and blood compatibility after prolonged use. We are currently in the process of conducting these tests.

## 5. Materials and Methods

### 5.1. Bacterial Test Strain

*S. aureus* (ATCC^®^ 29213) was used for both in vitro and in vivo experiments. The minimum inhibitory concentration (MIC) and minimum bactericidal concentration (MBC) required to kill 99.9% of bacteria for minocycline, rifampicin, and minocycline and rifampicin in a 1:1 (*w*/*w*) mixture (M/R) against *S. aureus* were determined. MIC and MBC assays were carried out in 96-well microtiter plates with standard concentrations (ranging from 0.44 µg/mL for pure antibiotics and 0.22 µg/mL for M/R to 250 µg/mL) dissolved in phosphate-buffered saline (PBS) and diluted 1:1 in 2× concentration LB medium followed by inoculating with *S. aureus* in a 10:1 ratio (inoculum was prepared in PBS to a concentration of approximately 1 × 10^7^ colony-forming units (CFU) per mL). The microtiter plate was incubated at 37 °C for 24 h with shaking at 180 rpm. MIC was determined through visual inspection and MBC was determined by diluting and plating the supernatants to estimate CFU.

### 5.2. Antibiotic Loading of IPN Materials

Poly(2-hydroxyethyl methacrylate)-co-poly(ethylene glycol) methyl ether acrylate (pHEMA-co-PEGMEA) IPN material with a content of 24% pHEMA-co-PEGMEA hydrogel, kindly provided by Biomodics ApS, was used throughout the study. The production of the IPN material was described in our previous paper [14]. In brief, silicone elastomers are swelled in supercritical CO_2_ in a reactor followed by the addition of monomers. These are allowed to polymerize inside the silicone, forming the resulting IPN in a sequential process [18]. Excess monomers were removed by soaking the material in 96% ethanol for 7 days followed by drying at 50 °C until the final weight was reached.

The IPN material was cut into four rectangular and circular patches measuring 5 mm × 10 mm × 2 mm and 12 mm in diameter × 0.2 mm, respectively, for in vitro experiments. Rectangular patches had a material weight of 148 ± 8.37 mg. For in vivo experiments, the IPN material was cut into rectangular patches measuring 20 mm × 10 mm × 0.6 mm. The IPN material was previously described by Klein et al. [14]. The IPN patches were sterilized in 96% ethanol and air-dried before incubation in 10 mg/mL rifampicin diluted in 96% ethanol for 7 days at room temperature. Subsequently, the same IPN patches were incubated for 7 days in 10 mg/mL minocycline diluted in PBS. Drug loading of the IPN patches for the animal experiments was conducted as described above. However, for the in vivo study, the second loading of 10 mg/mL minocycline solution was prepared in MilliQ water saturated with 2.5 mg/mL rifampicin to account for equilibrium drug release. Minocycline- and rifampicin-loaded IPN patches are hereafter denoted as M/R IPN patches.

### 5.3. Drug Release Quantification

Rectangular M/R IPN patches loaded with minocycline and rifampicin were placed in Eppendorf tubes containing 1 mL PBS and incubated overnight in the dark. M/R IPN patches were rinsed daily with PBS and transferred to new Eppendorf tubes containing 1 mL PBS. The tubes containing drug release medium were immediately frozen and stored at −18 °C until further analysis. Drug release was measured for 16 days in quadruplicates. A UV-vis spectrum ranging from 190 nm to 600 nm for rifampicin, minocycline, and M/R at a concentration of 15.66 µg/mL in PBS for each drug was recorded using a BMG SPECTROstar Nano spectrophotometer. From the UV-vis spectra, two suitable wavelengths for determining the concentration of minocycline (at 245 nm) and rifampicin (at 475 nm) in the drug release medium were identified. Minocycline and rifampicin are known to have overlapping spectra [29]; thus, the two selected wavelengths represent local absorption extrema, which are required to calculate the drug concentration at overlapping absorbances. Standard curves to calculate the unknown concentration of antibiotics in the release medium were generated by recording known antibiotic concentrations ranging from 1.95 µg/mL to 250 µg/mL in PBS. Drug release media were thawed and quantified by measuring the absorbances at both 245 nm and 475 nm using 96-well UV-Star^®^ microplates (cat. no. 655801, Greiner Bio-One, Kremsmünster, Austria,).

### 5.4. Functional Release Assay

The efficacy of the antibiotic release medium was tested against *S. aureus* in a functional release assay. The assay was performed according to the protocol for the MIC assay elaborated above. In short, drug release medium was supplemented with 2× concentration LB medium in a 1:1 solution and inoculated with *S. aureus* (approx. 1 × 10^7^ CFU/mL) in a 10:1 ratio. The plate was incubated at 37 °C for 24 h with shaking at 180 rpm. The final surviving *S. aureus* bacteria were compared to the *S. aureus* inoculum added to the LB/release medium. MIC was determined through visual inspection, and MBC was determined by diluting and plating the growth medium to estimate CFU.

### 5.5. Agar Disc Diffusion Assay

*S. aureus* was adjusted to McFarland 0.5 (wavelength 625 nm) in 0.9% saline and uniformly seeded on LB agar plates (Art. no. 98833, SSI Diagnostica, Hillerød, Denmark) using a cotton swab and plate spinner. The circular M/R IPN patches were placed in the middle of the agar plates and incubated overnight at 37 °C. After 24 h (+/−1), the inhibition zone diameters were measured with a ruler at two different sites. The same IPN patches were transferred to new plates for subsequent incubation. The experiment was performed in four replicates over 16 days.

### 5.6. Porcine Tissue Implantation Model

A porcine tissue implantation model was established and used to test the antimicrobial properties of M/R IPN patches versus heparin-coated ePTFE material (GORE^®^ PROPATEN^®^, Gore Medical, Flagstaff, Arizona). The porcine model consisted of 6 implants on each backside of the pigs, with either M/R IPN patches or heparin-coated ePTFE patches.

Prior to implantation, the M/R IPN patches were washed for 15 min in PBS to eliminate potential effects of surface-adsorbed antibiotics. The pigs were anesthetized with a mixture of zolamine (5 mg mL^−1^), butorphanol (1 mg mL^−1^), ketamine (10 mg mL^−1^), and xylazine (2 mg mL^−1^), dosing 1 mL of mixture per 15 kg bodyweight. The pigs were then placed on their abdomen and prepared for antiseptic operation. Six incisions of 3 cm on both sides of the back with 10 cm spacing were performed down to the fascia of the underlying muscle (Figure 4). M/R IPN patches were placed on the right side, while ePTFE patches were placed on the left side.

In the preliminary experiment, *S. aureus* doses increasing 10-fold from 10^3^ to 10^7^ CFU were inoculated at incision sites in six pigs after the placement of the patches (Figure 4). One additional incision site per CFU dilution series was mock-inoculated with sterile PBS as negative control. Based on the results from the preliminary experiment, a second experiment in two pigs was performed using fixed inoculation doses of 10^6^ CFU, as this yielded significant infections in all sites implanted with ePTFE patches. The overlying tissue of each pocket was adapted with consecutive 2-0 Vicryl suture, while the skin was closed with 0 Prolene as individual sutures. The cicatrices were sprayed with liquid plaster (OpSite, Smith & Nephew, Watford, United Kingdom) to prevent the pig from accessing the operative field. The experiments were terminated 2 weeks post-operation. Each skin pocket was opened, and the patches were removed and placed in tubes containing PBS + 0.1% Triton X-100 to release biofilm-associated bacteria. All tubes were then sonicated for 5 min followed by vortexing and plating for CFU quantification using serial dilutions. In the second experiment involving only one inoculation concentration, the area of each patch was also swabbed (eSwab^®^, Copan, Brescia, Italy).

### 5.7. Statistical Analysis

Viable bacterial count data from IPN patches were converted to CFU/mL. One-way ANOVA with Tukey’s Honest Significant Difference test or Mann–Whitney unpaired t-test was used for statistical analysis. The data are presented in the figures as means ± standard deviation. *p*-values < 0.05 were considered significant. Microsoft Excel (version 16.75) was used for transforming raw data, and GraphPad Prism 9 (version 9.5.1) was used for illustrations and analyses.

## Data Availability

The data presented in this study are openly available in Zenodo at DOI: 10.5281/zenodo.8355293.

## Supplementary Materials

The following supporting information can be downloaded at https://www.mdpi.com/article/10.3390/gels9100826/s1, Figure S1: UV-vis spectra for standard drug concentrations; Figure S2: Percentual daily drug release; Figure S3: Minimum inhibitory concentration assay; Figure S4: Agar disc diffusion assay; Figure S5: Agar disc diffusion assay—controls.
