# Supplementary material for: An Antibiotic-Loaded Silicone–Hydrogel Interpenetrating Polymer Network for the Prevention of Surgical Site Infections
Source: Gels. 2023 Oct 19;9(10):826. doi: 10.3390/gels9100826 (PMC10606314; doi:10.3390/gels9100826)
Supplement: Supplementary file 1 [file gels-09-00826-s001.zip › gels-2645161-supplementary.pdf]

Supplementary Materials

Figure S1: UV-vis spectra for standard drug concentrations

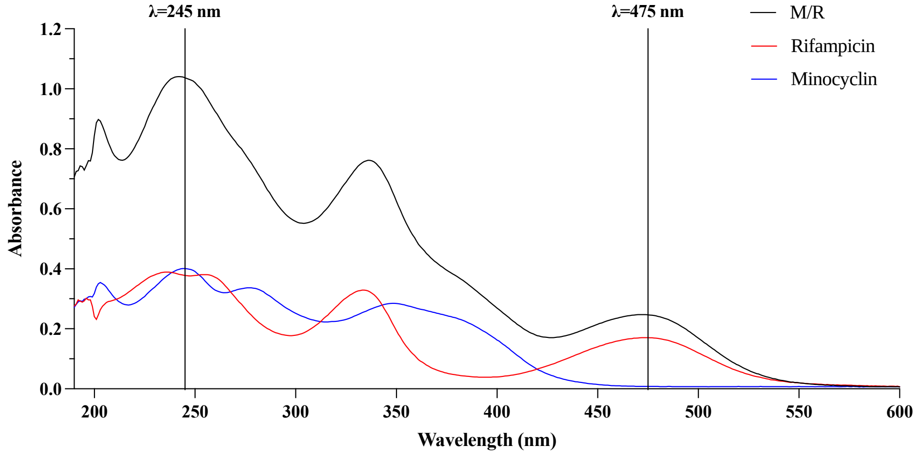

(a)

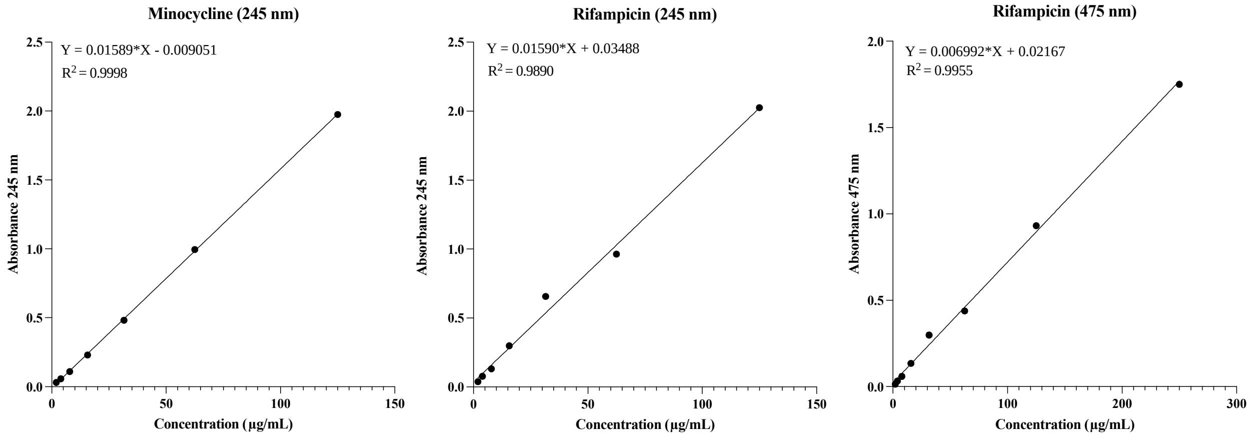

(b)

Figure S1. (a) UV-vis spectra ranging from 190 to 600 nm for pure minocycline and rifampicin and a mix of minocycline and rifampicin (M/R) in a 1:1 (15.625μg/mL w/w) mixture (black), rifampicin (red), and minocycline (blue). Wavelengths used for parallel quantifications are illustrated at λ=245 nm and λ=475 nm; (b) Calibration graphs of known minocycline and rifampicin concentrations aligned with the measured intensities of minocycline at 245 nm (left), rifampicin at 245 nm (middle) and rifampicin at 475 nm (right).

Figure S2: Percentual daily drug release

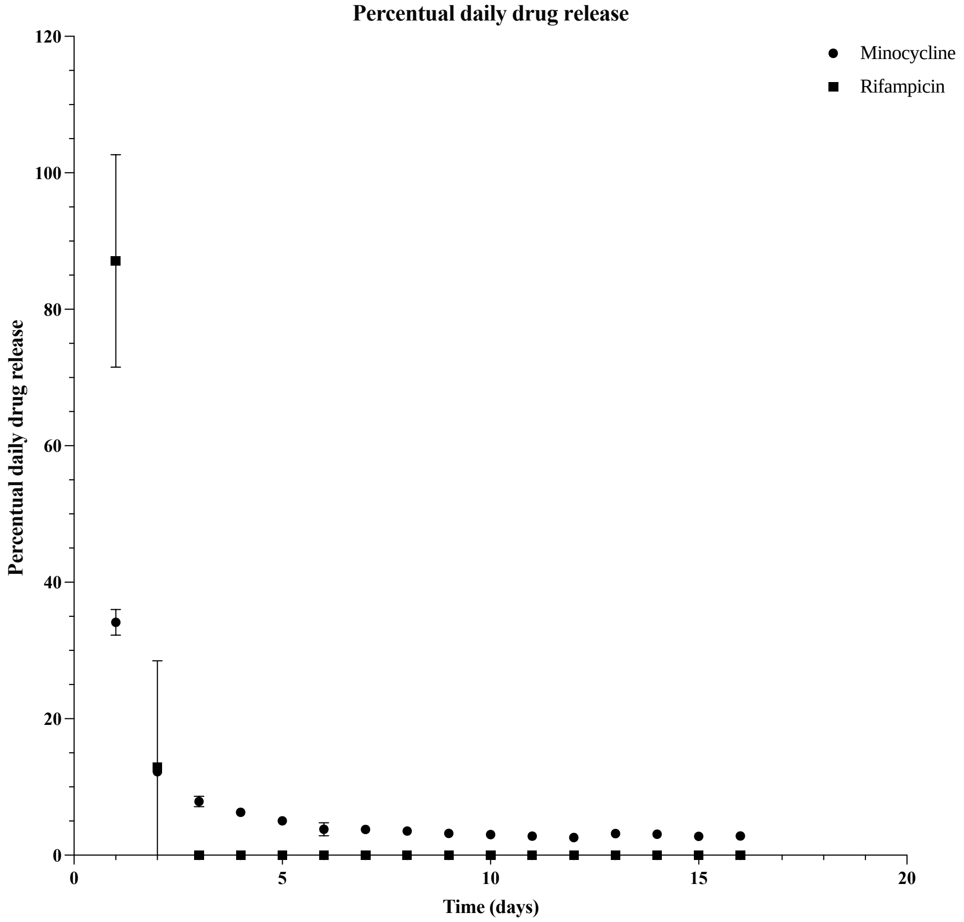

Figure S2: Percentual daily drug release of minocycline and rifampicin from the IPN material. Data are shown as means of four samples ± standard deviation over a time span of 16 days.

Figure S3: Minimum inhibitory concentration assay

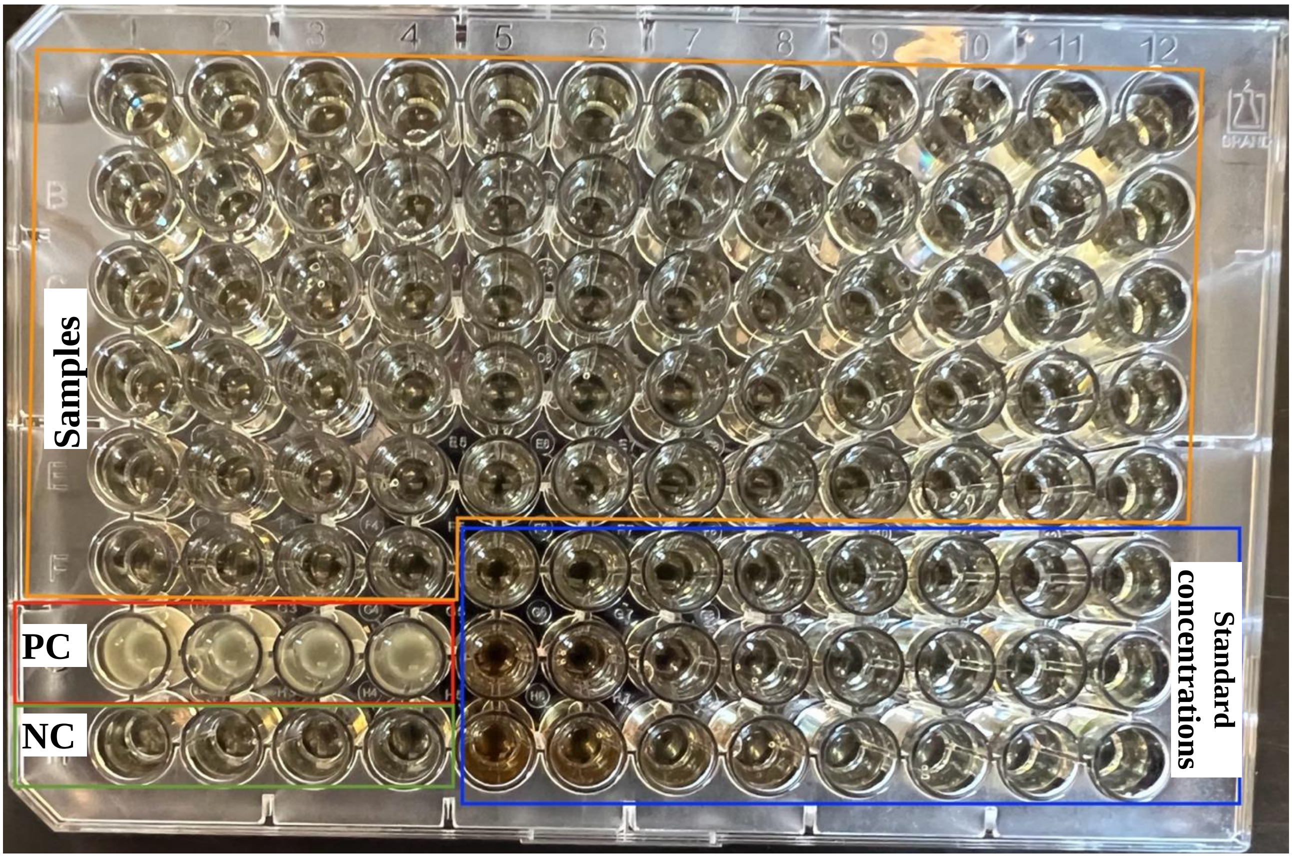

Figure S3. MIC assay of drug release medium against *S. aureus* (ATCC® 29213). Abbreviations; PC = positive controls, NC = negative controls.

Figure S4: Agar disc diffusion assay

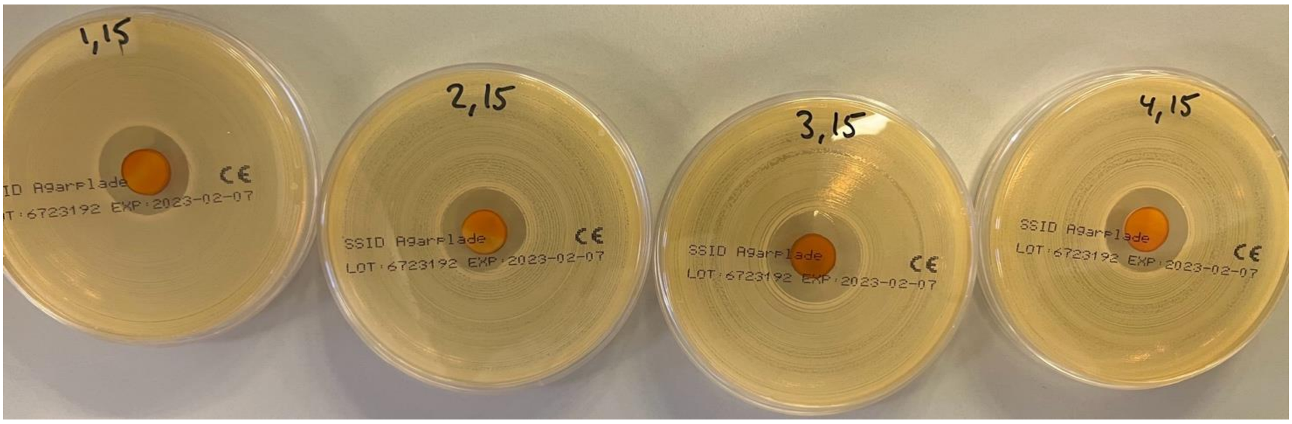

Figure S4. The inhibiting effect near the surface of the antibiotic loaded IPN patches against *S. aureus* (ATCC® 29213). Inhibition zones of loaded IPN patches after 15 days of release are shown.

Figure S5: Agar disc diffusion assay – controls

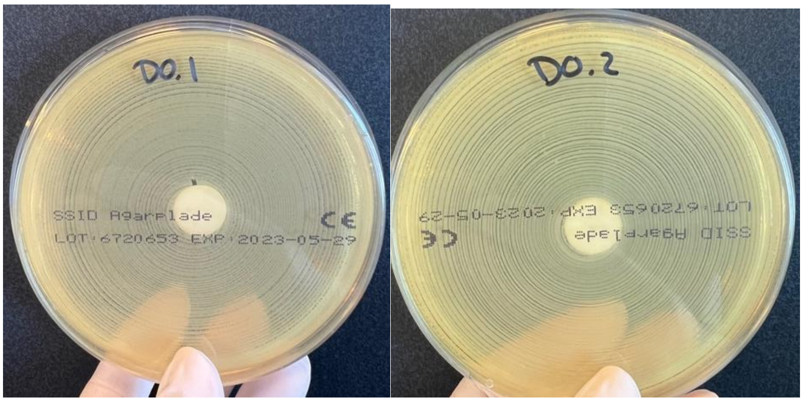

Figure S5. The inhibiting effect of non-loaded IPN patches against *S. aureus* (ATCC® 29213). IPN patches after 1 day are shown.
